# Care and Health Needs of Older Patients with Frailty in General Practice

**DOI:** 10.3390/healthcare14040518

**Published:** 2026-02-18

**Authors:** Katarzyna Wdowiak, Elżbieta Kozak-Szkopek, Anna M. Imiela, Andrzej Silczuk, Agnieszka Skowerska, Łukasz Czyżewski

**Affiliations:** 1 Department of Internal Diseases and Cardiology, Centre for Management of Venous Thromboembolic Disease, Medical University of Warsaw, 02-005 Warsaw, Poland; katarzynabielska@gazeta.pl (K.W.); elzbieta.kozak-szkopek@wum.edu.pl (E.K.-S.); anna.imiela@wum.edu.pl (A.M.I.); 2 Primary Health Care, Sobieski Medical Center, 02-936 Warsaw, Poland; 3 Department of Geriatric Nursing, Medical University of Warsaw, 02-007 Warsaw, Poland; 4 Department of Community Psychiatry, Medical University of Warsaw, 02-353 Warsaw, Poland; andrzej.silczuk@wum.edu.pl (A.S.); agnieszka.skowerska@wum.edu.pl (A.S.)

**Keywords:** frailty, older adults, health needs, primary care

## Abstract

**Background/Objectives**: Frailty is a major geriatric syndrome encountered in general practice. This study described the distribution of Clinical Frailty Scale (CFS) categories, care needs, and primary care utilization in a Vulnerable Elders Survey (VES-13)- screened high-risk cohort (VES-13 ≥ 3) from a single general practice. These prevalence estimates apply to this high-risk, single-centre primary care sample and should not be interpreted as estimates for unselected older primary care populations. **Methods**: We retrospectively reviewed medical records of 150 patients aged ≥ 60 years from a single primary care practice in Warsaw, Poland (1 August 2022–1 August 2023), restricted to those with VES-13 scores ≥ 3 (a routinely screened, high-risk subgroup). Frailty was assessed using the CFS. **Results**: The mean age of participants was 77 ± 8 years, and men accounted for 28% of the sample. Within this VES-13-selected high-risk cohort, 39 individuals (26%) were classified as non-frail (CFS 1–3), 72 (48%) as vulnerable (CFS 4), and 39 (26%) as frail (CFS ≥ 5). The need for assistance increased markedly with frailty severity, affecting 13% of non-frail individuals, 78% of vulnerable participants, and 100% of frail patients (*p* < 0.001). In the CFS ≥ 5 group, 46% required help several times per day and 8% required 24 h care. Patients with higher CFS scores used primary health care (PHC) services more frequently (mean 10 ± 5 visits per year in the non-frail group vs. 12 ± 6 in the vulnerable group and 17 ± 10 in the frail group; *p* < 0.001). **Conclusions:** In this single-practice, VES-13-selected high-risk primary care cohort, frailty (CFS ≥ 5) was observed in approximately one in four patients and vulnerability (CFS 4) in approximately one in two. Greater CFS severity was associated with higher care needs and more frequent primary care utilization.

## 1. Introduction

Population aging in Poland has accelerated in recent decades, with a marked increase in the proportion of adults aged ≥ 80 years and a substantial rise in primary care consultations among older adults [1,2]. As a result, general practice increasingly manages patients with multimorbidity and major geriatric syndromes, including frailty.

Frailty is a multidimensional clinical syndrome characterized by reduced physiological reserve and diminished resistance to stressors, leading to increased vulnerability to adverse outcomes [3,4,5]. It involves impairments across physical, nutritional, cognitive, and social domains and overlaps conceptually with disability, sarcopenia, and multimorbidity [6,7,8]. Frailty is consistently associated with falls, hospitalization, institutionalization, and mortality [9]. Its prevalence increases with age and is higher in women, as shown in both landmark cohort studies and meta-analyses [10,11,12,13,14,15].

Several instruments are used to assess frailty in clinical practice [16]. The frailty phenotype described by Fried et al. focuses on physical criteria [10], while the Edmonton Frail Scale incorporates multidomain assessment [17,18]. In primary care, the Clinical Frailty Scale (CFS) offers a rapid, observation-based classification linked to baseline functional status and levels of independence [19,20]. The CFS distinguishes nine levels of severity, allowing for pragmatic stratification into non-frail, vulnerable, and frail categories.

Community-dwelling older adults under primary care supervision are functionally heterogeneous. The Vulnerable Elders Survey-13 (VES-13) is a brief screening instrument identifying individuals at increased risk of functional decline and mortality [21,22]. A score ≥ 3 is commonly used to define a high-risk subgroup warranting further geriatric assessment. Linking such initial screening with frailty stratification may provide actionable information for triage and follow-up planning in routine practice [23]. Although frailty has been widely studied, evidence is limited on how CFS strata map onto practical care needs and primary care utilization in routinely screened high-risk patients.

The primary objective of this study was to compare care needs, including assistance requirements and mobility support, as well as primary care utilization, across CFS strata in a VES-13-selected high-risk primary care cohort. A secondary objective was to examine whether patients classified as vulnerable (CFS 4) demonstrate a care need and utilization profile closer to frail (CFS ≥ 5) or non-frail (CFS 1–3) individuals, thereby informing preventive targeting and follow-up intensity in routine primary care.

## 2. Materials and Methods

This retrospective single-centre study analyzed medical records of patients attending one primary care practice in Warsaw, Poland, between 1 August 2022 and 1 August 2023. The study group comprised patients aged 60 years and older who scored 3 points or more on the Vulnerable Elders Survey 13 (VES-13), indicating a risk of functional decline [21]. VES-13 was administered routinely during medical visits in the practice, and the total score was recorded in the medical record as part of standard geriatric risk screening. For this study, we extracted the documented VES-13 total scores and used VES-13 ≥ 3 as the inclusion criterion; VES-13 was not calculated retrospectively by the research team. Medical records were reviewed, and data were abstracted by a family physician (first rater). CFS ratings and extracted variables were independently verified by a second clinician (geriatrician; second rater) using the same pre-specified abstraction framework; disagreements were resolved by consensus adjudication. Formal inter-rater agreement statistics (e.g., kappa) were not calculated; however, independent dual rating followed by consensus adjudication was used to minimize misclassification in retrospective CFS assignment. This manuscript is reported in accordance with the STROBE statement for observational studies.

Frailty status was assessed retrospectively using the CFS based on documented baseline function in the medical record. CFS ratings were derived from: (1) documented ADL and IADL function; (2) mobility status (independent ambulation vs. cane/walker vs. wheelchair vs. bedbound); (3) use of assistive devices; (4) continence-related care needs when recorded (e.g., use of absorbent products); and (5) documented need for assistance and its frequency based on patient/family reports recorded during visits and/or formal care-related orders/referrals. To avoid capturing transient functional decline during acute illness, ratings were anchored to the patient’s usual (baseline) function as described in routine notes and home-visit documentation rather than short-term deterioration episodes. When documentation was insufficient to assign a higher category with confidence, a conservative lower rating consistent with the available baseline function was assigned. CFS categories were assigned retrospectively from medical records using published descriptors and a pre-specified abstraction framework: CFS 1–3 indicated independence in ADL/IADL, CFS 4 indicated independence in basic ADL with documented slowing/activity limitation, and CFS ≥ 5 indicated a need for assistance with IADL and/or basic ADL. The CFS includes 9 frailty levels: level 1 includes individuals who are healthy and very active; level 2 includes individuals in good physical condition; level 3 includes individuals with well-controlled medical problems who are occasionally active; level 4 represents a transitional stage (hereafter termed “vulnerable”) in which individuals are not dependent on others for daily help, but symptoms often limit activities and they are typically “slowed up”; level 5 corresponds to mild frailty and includes individuals requiring assistance with IADL; level 6 corresponds to moderate frailty and includes individuals requiring assistance with ADL and IADL; level 7 indicates severe frailty and includes individuals dependent on caregivers but clinically stable; level 8 indicates very severe frailty and includes individuals dependent on caregivers and approaching end of life; and level 9 includes terminally ill individuals with an expected survival of less than 6 months. For the purposes of this study, participants were classified into three groups according to the CFS: non-frail (CFS 1–3), vulnerable (CFS 4), and frail (CFS ≥ 5).

From the medical records, we extracted age, sex, living arrangement (living alone, living with spouse/partner, living with children/grandchildren, living with a caregiver), mobility (independent ambulation, cane/walker use, wheelchair use, bedbound), and use of absorbent incontinence products. We also extracted care-need variables. Need for assistance was coded as present only when the record explicitly documented dependence on another person for ADL and/or IADL tasks and/or receipt of informal or formal caregiving support (as described in routine visit notes, home-visit notes, patient/family reports recorded during encounters, and/or care-related orders/referrals for nursing or caregiving services). Assistance frequency was categorized as: once per week, several times per week, once per day, several times per day, or continuous (24 h), according to the description in the record. If assistance was documented but frequency was not specified, assistance frequency was coded as missing. Primary care utilization was assessed as the number of physician consultations recorded for each included patient during the study period. Subsequently, comparative analyses were performed according to frailty status, and the results were subjected to statistical analysis.

### 2.1. Ethics Statement

The study was approved by the Research Ethics Committee at the Medical University of Warsaw (No. AKBE/280/2023). Due to the retrospective design and the use of anonymized data, the requirement for informed consent was waived. The research was conducted in accordance with the Declaration of Helsinki, Good Clinical Practice, and the General Data Protection Regulation (GDPR).

### 2.2. Statistical Analysis

Results are presented as mean and standard deviation (SD) for continuous variables and as absolute numbers and percentages for categorical variables. Normality was assessed using visual inspection (histograms and Q–Q plots) and the Shapiro–Wilk test. As the distribution of continuous variables deviated from normality, comparisons between the three CFS groups (non-frail, vulnerable, frail) were performed using the Kruskal–Wallis analysis of variance (ANOVA) by ranks. Post hoc pairwise comparisons were conducted using the Mann–Whitney U test with Bonferroni’s correction. Correlations between CFS and selected continuous variables were assessed using Spearman’s rank correlation. Categorical variables were compared using the chi-squared test, and the strength of association was expressed using Cramér’s V. For 2 × 2 tables, odds ratios (OR) with 95% confidence intervals (CI) were calculated, and Fisher’s exact test was applied when expected cell counts were small. To examine variables independently associated with frailty classification (CFS ≥ 5), multivariable logistic regression was performed with age, sex, and annual PHC visit count as explanatory variables. The model was intentionally parsimonious given the limited number of frailty events (*n* = 39). Age and sex were included a priori as fundamental demographic correlates of frailty, while PHC visit count was included as a pragmatic utilization marker to test whether care contact frequency remained associated with frailty classification beyond demographics in this high-risk cohort. Several clinically relevant confounders, including multimorbidity burden, polypharmacy, socioeconomic status, and cognitive impairment, were not included because they were not available in a sufficiently standardized form in the retrospective records and/or would have exceeded feasible model complexity. Results are reported as ORs with 95% CI and corresponding *p*-values. Missing data were minimal and limited to use of absorbent incontinence products (*n* = 1/150; 0.7%); all other core variables (age, sex, living arrangement, mobility, need for assistance, PHC visit count, and CFS group) were complete. Because this was a retrospective study including all eligible patients within the study period, no a priori sample size calculation was performed. Sample adequacy was assessed primarily in terms of precision, and we report 95% confidence intervals (CIs) for key prevalence and proportion estimates. We did not calculate a posteriori (post hoc) power; instead, we emphasize effect estimates with 95% confidence intervals, which are more informative for interpreting precision in observational data. For regression analyses, the number of frailty events (*n* = 39) limited model complexity; therefore, multivariable logistic regression was restricted to three covariates (events per variable = 13), and regression results are interpreted as exploratory associations. All analyses were performed using Statistica 13.0 (StatSoft, Tulsa, OK, USA). A *p*-value below 0.05 was considered statistically significant.

## 3. Results

### 3.1. Characteristics of the Study Group

A total of 150 PHC patients aged 60 years and older were included. The mean age was 77 ± 8 years, and men accounted for 28% of the sample. According to the CFS, 39 participants (26%) were classified as non-frail (CFS 1 to 3), 72 (48%) as vulnerable (CFS 4), and 39 (26%) as frail (CFS 5 or higher) (Table 1). No statistically significant differences in sex distribution were observed between the groups (*p* = 0.109).

### 3.2. Profile of the Vulnerable Group

Within this VES-13-selected high-risk cohort, vulnerable participants (CFS 4; *n* = 72) already showed substantial support needs despite preserved basic ADL independence. A documented need for assistance was present in 78% (56/72). Among those requiring help (*n* = 56), assistance was most commonly provided several times per week (68%; 38/56) or once per week (20%; 11/56), whereas daily assistance was less frequent (once per day: 4%; 2/56; several times per day: 9%; 5/56); none required continuous (24 h) care. Early mobility limitation was also evident: 17% (12/72) used a cane or walker, and 46% (33/72) lived alone. This pattern suggests that patients classified as vulnerable (CFS 4) may benefit from low-threshold, prevention-oriented interventions in routine primary care.

### 3.3. Clinical and Functional Parameters

With increasing frailty severity, mean age differed across CFS strata (72 ± 6 vs. 77 ± 7 vs. 83 ± 7 years; Kruskal–Wallis *p* < 0.001). Bonferroni-adjusted post hoc comparisons confirmed differences between all pairs (non-frail vs. vulnerable: *p* < 0.001; vulnerable vs. frail: *p* < 0.001; non-frail vs. frail: *p* < 0.001).

Primary care utilization also differed across CFS groups (10 ± 5 vs. 12 ± 6 vs. 17 ± 10 visits/year; Kruskal–Wallis *p* < 0.001). Bonferroni-adjusted post hoc comparisons showed higher visit counts in frail patients compared with both non-frail (*p* = 0.002) and vulnerable participants (*p* = 0.035), whereas non-frail and vulnerable participants did not differ (*p* = 0.241).

A clear gradient was observed for independence. All non-frail participants were able to walk independently, whereas the proportion decreased to 83% in the vulnerable group and to 41% among frail patients (*p* < 0.001). Use of a cane or walker was reported by 0%, 17%, and 59% of participants, respectively. Absorbent incontinence products were used by 13% of frail patients, with no such cases in the other groups (*p* < 0.001).

The need for assistance increased markedly with frailty severity. It was reported for 13% of non-frail individuals, 78% of the vulnerable group, and all patients with frailty (*p* < 0.001). Notably, 46% of participants with CFS 5 or higher required assistance several times per day, and 8% required continuous 24 h care. Because functional dependence and the need for assistance are embedded in the CFS definitions, associations between CFS strata and assistance-related variables should be interpreted as construct-consistent functional correlates rather than independent predictors of frailty.

### 3.4. Social Conditions

Living arrangement differed across CFS groups; however, cell counts were small in some categories, and findings should be interpreted cautiously. Frail patients more often lived with children or grandchildren (18% vs. 3% in the non-frail group; *p* = 0.045), which may reflect increasing care needs.

### 3.5. Correlation Analysis

**Figure 1:**
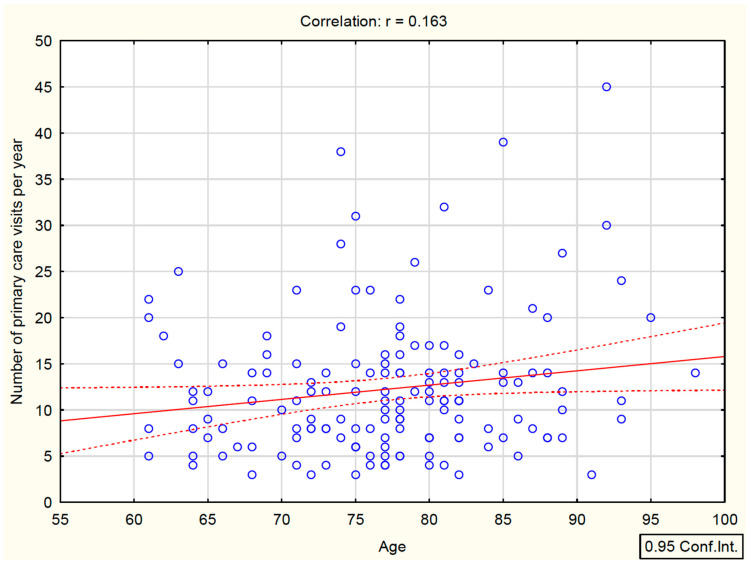
Correlation between age and the number of PHC visits per year.

A moderate positive correlation was found between age and CFS (r = 0.526, *p* < 0.05). In addition, the number of PHC visits per year correlated significantly with frailty level (r = 0.283, *p* < 0.05) (Table 2). Graphical analysis (Figure 1) suggested a weak positive association between age and the number of PHC consultations (r = 0.163, *p* < 0.05), with higher visit counts observed in many patients aged > 75 years.

### 3.6. Logistic Regression

In the multivariable model, variables that were independently associated with classification as frail (CFS ≥ 5) were age (OR = 1.16 per year; 95% CI 1.08 to 1.24; *p* < 0.001) and annual PHC visit count (OR = 1.10 per visit; 95% CI 1.04 to 1.17; *p* = 0.002). Sex was not statistically significant (*p* = 0.278) (Table 3).

### 3.7. Univariable Associations

In the univariable analyses, need for assistance showed the strongest association with classification as frail (OR = 63.9; 95% CI 3.8 to 1066.7; *p* < 0.001). However, this should be interpreted as a construct-consistent functional correlate because assistance is conceptually embedded in the CFS descriptors. Assistance several times per day (OR = 18.2; 95% CI 6.1 to 54.4) and cane/walker use (OR = 11.9; 95% CI 4.9 to 28.5) were also strongly associated with frailty classification (all *p* < 0.001) (Table 4).

## 4. Discussion

This study focuses on a selected high-risk population of older adults in PHC. It included patients aged ≥ 60 years with VES-13 scores ≥ 3, indicating increased risk of functional decline. Frailty assessed with the CFS was present in approximately one in four participants, while nearly one in two were classified as vulnerable (i.e., at risk of frailty). We observed that frailty severity increased with age. In the Polish population, based on data collected within the PolSenior2 project [24] involving nearly 6000 participants, frailty was identified using the Fried criteria in 15.9% of community-dwelling adults aged 60 years and older. Its prevalence increased markedly after the age of 70 years.

Beyond estimating frailty prevalence, our results add practice-relevant detail by showing how quickly assistance needs rise already at CFS 4 within a routinely screened high-risk cohort. This extends routine primary-care frailty assessment by quantifying an explicit care-need and utilization gradient across CFS categories within a routinely screened, VES-13-selected high-risk cohort, which can inform follow-up intensity and preventive targeting. Given the retrospective observational design, this study does not support causal inference or prescriptive care pathways. Rather, the observed gradients in assistance frequency and primary care utilization can be used as pragmatic signals to inform local triage and follow-up planning in general practice but require prospective validation before being used as decision rules. A brief mechanistic interpretation supports these descriptive gradients. Frailty is commonly understood as progressive loss of physiological reserve across multiple systems (e.g., musculoskeletal, immune, endocrine and neural), which reduces homeostatic capacity and amplifies the functional impact of relatively minor stressors. As reserve declines, patients experience disproportionate fatigue, slowed gait, impaired balance and reduced strength, translating into stepwise loss of IADL capacity and, at higher severity, dependence in basic ADL—clinically manifesting as recurrent need for caregiving assistance and (in more severe frailty) higher healthcare contact. This framework helps interpret the observed rise in assistance intensity across CFS categories as functional consequences of reserve depletion rather than isolated correlational findings [4,25].

Across European countries included in the Survey of Health, Ageing and Retirement in Europe (SHARE) [26], frailty assessed using the Global Burden of Disease Frailty Index (GBD-FI) was present in an average of 22% of participants, ranging from 8% in Switzerland to 41% in Poland, and it increased with age. A similar age-related increase in frailty prevalence has also been reported by Asian researchers [27].

As frailty progresses, care needs increase. In the present study, all patients with frailty required assistance, and almost one in two needed help several times per day. Importantly, the vulnerable group already showed substantial support needs: most received assistance several times per week or weekly, while daily or more frequent assistance was uncommon (about 10% of the full CFS 4 group). The need for assistance was also reflected in the more frequent co-residence of frail individuals with family members, which underscores that frailty assessment in routine practice should address not only the medical dimension but also the practical conditions that enable care to be obtained and sustained, including family support, transport, and service availability.

At this point, it is important to distinguish between the severity of health needs and actual service use. Increasing needs among frail individuals do not always translate into proportionate use of care, because access may be modified by systemic and environmental barriers. In the IN-AGE project [28], conducted in three regions of Italy using a mixed-methods design with a dominant qualitative component, key obstacles included long waiting times in the public sector, the cost of private services, and distance to facilities, which created a need for transport and an accompanying person. Waiting times were reported by both sexes, slightly more often by men. Transport and distance barriers concerned almost exclusively women, and in the private sector, cost was primarily a barrier for women. In extreme situations this resulted in foregoing consultations, diagnostics, or therapy when the cost was prohibitive, the appointment was too distant, or travel without support was not feasible. Therefore, when assessing the care needs of frail patients in PHC, it is necessary to consider not only clinical status but also the patient’s functional access to the system, including transport, informal support, costs, and waiting times. These factors may affect adherence to recommendations and the risk of treatment delays.

Regardless of the access barriers described above, the literature consistently confirms a close relationship between frailty severity and functional status, which supports the inclusion of functional assessment in routine PHC practice. In the PolSenior2 study, older adults with frailty were significantly more likely to require ad hoc or regular help from others. Only 3.5% of frail individuals and 50% of vulnerable individuals did not require regular assistance [24].

The progression of mobility impairment with age leads to the need for support with transfers and ambulation. According to PolSenior2, 88.1% of adults older than 60 years did not require assistance with mobility, but the proportion of independently mobile individuals decreased with age and was below 40% among those aged 90 years and older. In younger age groups, a walking stick or support from another person was most commonly used, whereas after the age of 80 years, a walker was used more often [29]. Mobility mode correlates strongly with frailty severity. Chinese researchers described the use of mobility aids among older adults with frailty. Among vulnerable individuals, 69.5% walked independently, while among frail individuals, 69.5% used assistive devices. The most frequently used aid was a cane (44.7%), followed by a walker (41.1%) and a wheelchair (24.3%) [30]. Similarly, in our study, nearly 60% of frail patients used assistive equipment, most often a cane or walker.

Available evidence indicates that individuals with frailty use health care services more frequently. Thandi et al. [31] showed that patients with frailty had more prescribed medications and more visits to health care facilities.

In the present study, the frequency of physician consultations correlated with frailty severity and with age, particularly among patients older than 75 years. A frail patient attended an average of 17 physician visits over 12 months, that is, approximately 1.4 visits per month, typically one to two visits monthly. In contrast, patients without frailty attended an average of 10 visits, which corresponds to fewer than one visit per month. These findings indicate higher use of PHC consultations among frail patients. They do not, however, determine whether the care received is adequate to need, because contact frequency is also influenced by access barriers and the organization of service delivery. The observed association between PHC visit frequency and frailty should be interpreted cautiously because several unmeasured factors may drive both outcomes. In particular, multimorbidity burden and polypharmacy typically increase clinical monitoring and service use while also co-occurring with frailty; cognitive impairment may independently increase both functional vulnerability and healthcare contacts; and socioeconomic status can influence both baseline health and access patterns. Omission of these covariates may have inflated (or, less likely, attenuated) the estimated association between PHC utilization and frailty, and therefore, the regression findings should be regarded as exploratory and hypothesis-generating rather than causal.

Frail patients may also more often require institutional care. Chinese investigators reported an association between frailty and the need for long-term care, and their modelling analysis suggested more than a threefold higher demand for long-term care among frail individuals compared with non-frail individuals [32]. Particular attention should be paid to individuals at risk of frailty, as they may benefit from preventive interventions. In our study, vulnerable individuals accounted for nearly 50% of the high-risk group (VES-13 ≥ 3 points). Almost one in two vulnerable individuals lived alone; 17% reported mobility problems and required support with a cane or walker. More than three-quarters of vulnerable individuals reported needing help with everyday functioning, and 78% of vulnerable individuals received assistance once a week or more often. Overall, vulnerable individuals were characterized by living alone, early use of mobility aids, and substantial need for assistance. Individualized preventive interventions should therefore aim to improve or maintain independence. This is clinically relevant, as vulnerability is commonly conceptualized as a transitional (potentially modifiable) state that may precede progression to frailty [33].

Accurate identification of frailty and of patients at risk in general practice is essential for planning and implementing interventions, both preventive and therapeutic. At present, there is no single diagnostic method for frailty that is accepted as a standard. In our study, we used the CFS because it is simple and quick to apply by caregivers, nurses, physiotherapists, and physicians [20]. The utility of the CFS in PHC was demonstrated in a Canadian study in which family physicians used the CFS to identify, among 2043 patients, 18.64% as vulnerable and 25.50% as frail. In that study, frailty was linked to social risk factors and chronic diseases to identify patients with the highest needs and to design targeted interventions [34].

In 2017, the National Health Service (NHS England) introduced proactive frailty identification into the contract for general practitioners. However, it was found that identification of frailty was largely ad hoc and opportunistic [35]. A survey among general practitioners in the United Kingdom indicates that clinicians in PHC consider trusted relationships with patients and the support of experienced clinicians to be important in the management of frailty [36].

From a care organization perspective, the observed clustering of needs, including multimorbidity, functional dependence, and more frequent PHC contacts, supports implementation of an integrated approach. This includes multidimensional geriatric assessment, coordination of care between the PHC physician and specialists, and attention to environmental components such as transport, caregiver support, and the availability of home visits. The authors of the IN-AGE study emphasize that reducing waiting times, improving transport availability, and lowering costs, particularly important for women with limited financial resources, are prerequisites for meaningful access to care for frail individuals. Without such measures, the risk of delays, missed care, and secondary complications of chronic disease may increase [28].

The literature also highlights the importance of preventing frailty and implementing preventive measures before the age of 60 years. These measures include healthy dietary habits, physical activity promotion, vitamin D3 supplementation, and systematic review of medications and supplements used by the patient. In a review, United Kingdom authors evaluated interventions implemented in older patients with established frailty in general practice. The analysis included 29 studies conducted between 2000 and 2019, most from the Netherlands and Spain, with nine other countries represented by one study each: Japan, China, Australia, Austria, Canada, France, the United States, Switzerland, and Mexico. Interventions were grouped into two categories, one focused on comprehensive assessment and treatment and the other targeting specific needs of people with frailty. The analysis suggests that patients valued interventions that matched their needs and capacities [37].

Early frailty screening, focused mainly on nutrition, functional status, physical activity, and social participation, aims to detect and prevent frailty at an early stage and to reduce its prevalence. This strategy enables targeted interventions, especially among vulnerable patients [27]. Given population ageing, frailty is expected to become one of the major public health challenges [25].

### 4.1. Implications for Public Health and Preventive Practice

The following considerations are intended as pragmatic implementation guidance derived from observational data rather than as a prescriptive algorithm. In this VES-13-selected high-risk primary care cohort, frailty severity was consistently associated with increasing assistance needs, while higher primary care utilization was observed mainly among frail patients. Importantly, service use may not fully reflect need, as it can be modified by functional access barriers such as mobility limitations, caregiver availability, transportation, waiting times, and cost.

A concise three-step approach for routine primary care may therefore be considered:

**Step 1:** Case finding. Use a brief screening tool feasible within routine visits, such as VES-13. Patients with scores ≥ 3 should undergo further geriatric assessment, including frailty evaluation.
**Step 2:** Frailty stratification. Assign a baseline Clinical Frailty Scale category anchored to usual function rather than acute deterioration. Document key functional markers relevant to care planning, including assistance needs and mobility status.
**Step 3:** Tiered management.

- CFS 1–3: continue standard chronic disease management with periodic reassessment.
- CFS 4: implement low-threshold multidomain preventive measures and schedule proactive follow-up, given the substantial assistance needs already observed at this stage.
- CFS ≥ 5: initiate structured, coordinated care planning, including caregiver support and collaboration with community and social services.

Routine assessment should explicitly consider functional access barriers to ensure that care delivery aligns with actual need. Prospective studies are required to determine whether this screening and stratification framework improves outcomes and equity of access in primary care.

### 4.2. Limitations of the Study

This study was retrospective and single-centre and relied on data from medical records, which limited control over completeness and standardization of information. The retrospective use of routine medical records introduces the risk of information bias. Several variables (e.g., living arrangement and the frequency of assistance) were based on patient or family reports documented during clinical encounters and may be affected by recall or reporting bias. In addition, retrospective CFS classification may be subject to interpretation bias and misclassification if documentation of baseline function was incomplete or heterogeneous across patients. To mitigate this, CFS ratings and extracted data were independently verified by a second clinician and discrepancies were resolved by consensus; nevertheless, residual misclassification cannot be excluded.

The sample included individuals aged 60 years and older with VES-13 scores of 3 or higher, that is, a population at increased risk of functional decline. Therefore, prevalence estimates should be interpreted as applying to older primary care patients with VES-13 scores ≥3 rather than to the entire primary care population aged ≥ 60 years. Some analyses involved functional variables, such as the need for assistance, that are conceptually close to the CFS construct, which may amplify observed associations and require careful interpretation.

The sample size and small numbers in some categories may have affected the precision of estimates, resulting in wide confidence intervals, and the findings should be considered exploratory; accordingly, we limited the multivariable model to three covariates to reduce overfitting risk given the number of frailty events. The multivariable logistic regression included only age, sex, and annual PHC visit count and did not adjust for several clinically important confounders, including multimorbidity burden, polypharmacy, socioeconomic status, or cognitive impairment. These factors may affect both frailty status and healthcare utilization; consequently, residual confounding is likely, and the magnitude of the association between PHC visit frequency and frailty may be overestimated. The regression results should therefore be interpreted as exploratory associations rather than evidence of independent or causal effects. Finally, the number of PHC visits reflects utilization rather than a direct measure of health need. Contact frequency may also be shaped by organizational factors and access barriers, so inferences about demand should be made cautiously. The study design does not allow for causal conclusions.

## 5. Conclusions

In this single-centre cohort of PHC patients aged ≥ 60 years with VES-13 scores ≥ 3 (a selected high-risk subgroup routinely identified in the practice), frailty was present in approximately one in four participants and vulnerability in approximately one in two. These prevalence estimates apply to this VES-13-selected high-risk primary care sample and should not be generalized to unselected older adults in primary care. Frail patients under the care of a general practitioner have greater care needs, more often require assistive mobility devices, and require more frequent physician consultations. For assessing the risk of frailty in older patients in general practice, the CFS is a simple and useful tool. These findings support a pragmatic two-step workflow in routine primary care: initial risk screening using VES-13 followed by rapid CFS stratification to guide follow-up intensity. In practice, this approach distinguishes patients who may benefit primarily from low-threshold, multidomain preventive measures (CFS 4) from those requiring proactive, integrated management with caregiver and community-service coordination (CFS ≥ 5). Given the retrospective single-centre design, these implications should be interpreted as implementation-oriented signals that warrant prospective multicentre validation before broader benchmarking or policy adoption.

## Figures and Tables

**Table 1:** Clinical characteristics by Clinical Frailty Scale (CFS) group.

Variable	Non-Frail (CFS 1-3)	Vulnerable (CFS 4)	Frail (CFS 5 or Higher)	All	*p*-Value
N Age, years PHC visits per year	39	72	39	150	
	72 ± 6	77 ± 7	83 ± 7	77 ± 8	<0.001
	10 ± 5	12 ± 6	17 ± 10	12 ± 8	<0.001
Male sex, *n* (%)	16 (41)	17 (24)	9 (23)	42 (28)	0.109
Living arrangement, *n* (%)					0.045
Living alone	17 (44)	33 (46)	12 (31)	62 (41)	
Living with spouse or partner	21 (54)	36 (50)	20 (51)	77 (51)	
Living with children or grandchildren	1 (3)	3 (4)	7 (18)	11 (7)	
Mobility, *n* (%)					<0.001
Independent	39 (100)	60 (83)	16 (41)	115 (77)	
Cane or walker	0 (0)	12 (17)	23 (59)	35 (23)	
Absorbent incontinence products, *n* (%)	0 (0)	0 (0)	5 (13)	5 (3)	<0.001
Need for assistance, *n* (%)	5 (13)	56 (78)	39 (100)	100 (67)	<0.001
Frequency of assistance, *n* (%)					<0.001
Continuous (24 h)	0 (0)	0 (0)	3 (8)	3 (2)	
Several times per day	0 (0)	5 (9)	18 (46)	23 (15)	
Once per day	0 (0)	2 (4)	1 (3)	3 (2)	
Once per week	2 (40)	11 (20)	0 (0)	13 (9)	
Several times per week	3 (60)	38 (68)	17 (44)	58 (39)	

Note: Percentages for “frequency of assistance” are calculated among participants who required assistance.

**Table 2:** Correlations between Clinical Frailty Scale (CFS) and selected variables.

Variable	Spearman r
Age	0.526 *
PHC visits per year	0.283 *

* *p* < 0.05.

**Table 3:** Multivariable logistic regression: factors associated with frailty (CFS 5 or higher).

Variable	OR	95% CI	*p*-Value
Age (per 1 year) Sex (male vs. female) PHC visits per year (per 1 visit)	1.161	1.084–1.244	<0.001
	0.574	0.211–1.565	0.278
	1.103	1.037–1.174	0.002

**Table 4:** Univariable odds ratios: selected functional characteristics correlated with classification as frail (CFS ≥ 5).

Factor	OR	95% CI	*p*-Value
Need for assistance (yes = 1) Assistance several times per day Cane or walker use	63.934	3.832–1066.699	<0.001
	18.171	6.074–54.364	<0.001
	11.859	4.943–28.456	<0.001

## Data Availability

The raw data supporting the conclusions of this article will be made available by the authors on request.
